# Comparative transcriptomics identifies genes differentially expressed in the intestine of a new fast-growing strain of common carp with higher unsaturated fatty acid content in muscle

**DOI:** 10.1371/journal.pone.0206615

**Published:** 2018-11-05

**Authors:** Chengfeng Zhang, Shengyan Su, Xinyuan Li, Bing Li, Baojuan Yang, Jian Zhu, Weimin Wang

**Affiliations:** 1 College of Fisheries, Huazhong Agricultural University, Wuhan, PR China; 2 Key Laboratory of Genetic Breeding and Aquaculture Biology of Freshwater Fishes, Ministry of Agriculture; Freshwater Fisheries Research Center, Chinese Academy of Fishery Sciences, Wuxi, PR China; 3 Wuxi Fisheries College, Nanjing Agricultural University, Wuxi, PR China; Universite de Liege, BELGIUM

## Abstract

We have created a new, fast-growing strain of common carp with higher unsaturated fatty acid content in muscle. To better understand the impacts of gene regulation in intestinal tissue on growth and unsaturated fatty acid content, we conducted a comparative RNA-Seq transcriptome analysis between intestine samples of Selected and Control groups (and corroborated selected results by PCR). After eight weeks of cage culture, weight gain of the Selected group was 20.84% higher. In muscles of the control group, monounsaturated fatty acids (FAs) were more abundant, whereas polyunsaturated FAs were more abundant in muscles of the Selected group. In total, we found 106 differentially expressed genes (DEGs) between the two groups. Only the endocytosis pathway was significantly enriched in DEGs, with two upregulated genes: *il2rb* and *ehd1*. The latter is involved in the growth hormone/insulin-like growth factor (Gh/Igf) axis, which plays a key role in the regulation of growth in animals. *tll2*, which is known to be associated with intestinal regeneration, was extremely highly upregulated in both transcriptomic (infinite) and qPCR (610.70) analyses. Two of the upregulated genes are associated with the fatty acid metabolism, several genes are likely to be indicators of heightened transcription levels, several are associated with metabolic and developmental roles, several with neuronal functions (including two with vision), several with the immune system, and two downregulated genes with the development of vasculature. The higher growth rate of the Selected group is likely to be at least partially attributed to increased endocytosis efficiency and genetically-driven behavioural differences (higher aggression levels). There are some indications that this new strain might have slightly impaired immune responses, and a higher propensity for inherited diseases leading to sight impairment, as well for neurodegenerative diseases in general, but these indications still need to be confirmed.

## Introduction

Growth rate, regulated by environmental factors and genetics, is a primary trait of interest in selection programs of most cultured fish species due to its intrinsic link with productivity and profitability of aquaculture enterprises [1]. The common carp (*Cyprinus carpio* Linnaeus 1758) is probably the oldest and most common cultured fish species, with third largest total aquaculture production output globally. In 2014, the total global output was over four million tonnes (almost 10% of the global annual freshwater aquaculture production), producing the economic value of almost six billion dollars, with China producing around 70% of the total output [2].

Growth of skeletal muscles of fish is the primary topic of interest for the aquaculture industry [3]. Several growth-related features set the fishes apart from mammals: continuous accretion of muscle tissue results in the increase of body length and mass (albeit at a slowing rate) until mortality or senescence occur, both hyperplasia and hypertrophy contribute to muscle growth, and fishes predominantly accumulate functional protein (as opposed to storing the excessive energy as adipose tissue) [3–5]. Although some molecular aspects of growth in fish are relatively well-studied, such as the growth hormone—insulin-like growth factor I (GH-IGFI) axis of the neuroendocrine system [3,6–8], our understanding of the molecular regulation of growth in fish remains fragmentary [3,9].

Fatty acids are essential cellular components, whose composition is important for the nutritive value and taste of meat [10]. In humans, saturated FAs are risk factors for cardiovascular diseases, whereas unsaturated and especially polyunsaturated FAs, such as omega-3 and -6 (ω-3 and ω-6), have antiatherosclerotic effects and a number of other beneficial health effects [11,12]. Fish meat is generally the main source of polyunsaturated fatty acids (FAs) in human diet, and thus of remarkable significance in human nutrition [13,14].

The gastrointestinal tract acts as a selectively permeable barrier for dietary nutrients, electrolytes and water, while maintaining an effective defense against pathogens [15]. Regardless of anatomical and physiological differences (common carp is an agastric, omnivorous fish), teleost fishes are characterised by the presence of at least two intestinal segments: the first is responsible for the absorption of lipids and the second for pinocytotic uptake of macromolecules, including proteins [3,4]. Uptake of nutrients from food via the intestinal barrier is a prerequisite for growth, and impacts of different diets on intestinal transcriptome have been studied in fish [16,17]. However, individual differences in the efficiency of this process in fish, and particularly the genetic background thereof, remain almost completely unstudied.

The molecular control of growth in fish muscles is relatively well-understood [5,9], but molecular control of intestinal nutrient uptake, although likely to play a part in the growth rate [9], remains poorly understood. High-throughput RNA sequencing technology (RNA-Seq) has been used to study the molecular mechanisms underlying growth in bacteria [18], plants [19], mammals [20], and fish [21–26]. Furthermore, detailed RNA-Seq transcriptome analyses have been applied to study gene expression profiles in 19 different tissues of the common carp, but the analysis did not include intestinal samples [27]. Intestinal transcriptomes have been used to search for drug candidates [28], study regeneration mechanism [29], and genome polyadenylation [30], but (to our knowledge) none of previous transcriptome-based studies of fish growth have used intestine among the sampled tissues [21,22].

A new, fast-growing strain of common carp with higher unsaturated fatty acid content in the muscle was recently created by combining the best linear unbiased prediction and molecular markers-based breeding methods [31]. To better understand the overall gene expression levels in the intestine of common carp, as well as the impacts of gene regulation in intestinal tissue on the growth and unsaturated fatty acid content in the muscles of this important cultured fish species, we conducted a comparative RNA-Seq transcriptome analysis of intestinal tissues of the new strain and a control group. This study also aims to contribute to the understanding of the genetic basis of growth and unsaturated fatty acid content in fish and vertebrates in general.

## Results and discussion

### Growth performance and effects of selection on fatty acid composition of muscles

Growth performance test of 48 families showed that after eight weeks of cage culture experiment, weight gain of the Selected group was 20.84% higher than that of the Control group (Table 1). Fatty acid composition analysis showed that saturated FAs (SFA) were more abundant (albeit non-significantly) in the muscles of Control group (Table 1). Among eight SFAs, six were more abundant in the Control group, but only one significantly. Regarding the two that were higher in the Selected group (C:18 and C:20), only C:18 was significantly higher. Unsaturated FAs (UFA), however, were more abundant in the Selected group: among 15 UFAs, only four were more abundant in the Control group, and only C18:1 significantly. Among the remaining eleven UFAs, three (C20:4, C22:3 and C22:4) were significantly higher in the Selected group. Intriguingly, this difference was driven exclusively by polyunsaturated FAs (PUFA), as monounsaturated FAs (MUFA) were significantly more abundant in the Control group: three out of four were more abundant. Among the PUFAs, only one (C18:3 *n-3*) was more abundant in the Control group (Table 1).

**Table 1:** Effect of selection on growth and long-chain fatty acid (FA) composition (% of total FA) of muscles.

Growth/FA	Control group	Selected group
IBW	11.88±2.63	16.09±1.90
FBW	38.08±7.84^a^	63.83±5.66^b^
BWG	26.20±6.87^a^	47.74±4.96^b^
C12:0	0.0175±0.0016^1^	0.0145±0.0007
C14:0	0.6256±0.0305	0.5794±0.034
C15:0	0.163±0.0111^a^	0.1365±0.0043^b^
C16:0	20.443±0.4523	19.321±0.4607
C17:0	0.2525±0.0162	0.2478±0.0112
C18:0	6.6548±0.0458^b^	7.4656±0.1645^a^
C20:0	0.2143±0.0118	0.2151±0.009
C22:0	0.0815±0.008	0.0714±0.0035
C16:1	1.4435±0.1591	1.0863±0.103
C18:1	20.511±0.5896^a^	17.236±0.7601^b^
C20:1	1.0023±0.0495	1.003±0.0234
C22:1	0.241±0.0314	0.2033±0.0074
C18:2	25.674±1.141	25.998±0.6794
C18:3 *n*-6	0.3213±0.0268	0.3878±0.0229
C18:3 *n*-3	1.5308±0.0725	1.4283±0.0453
C20:2	1.1575±0.0349	1.2028±0.0497
C20:3	2.398±0.0805	2.4934±0.0572
C20:4	3.9213±0.2575^b^	5.3394±0.2921^a^
C20:5	1.315±0.0422	1.3968±0.093
C22:3	0.3418±0.0152^b^	0.4541±0.0267^a^
C22:4	1.6068±0.0451^b^	2.3871±0.0909^a^
C22:5	0.8765±0.0422	0.9205±0.0391
C22:6	9.1105±0.2154	10.331±0.4868
SFA^2^	28.549±0.4591	28.132±0.3424
MUFA^3^	23.198±0.7656^a^	19.529±0.8618^b^
PUFA^4^	48.254±0.9015^b^	52.339±1.0159^a^
UFA^5^	71.452±0.4591	71.868±0.3424

IBW = initial body weight (g), FBW = final body weight (g), BWG = body weight gain (%). Within a row, different letters (superscript) indicate that the means are significantly different between two groups (*P*<0.05).

^1^ All values are presented as mean % ± SE

^2^ SFA = sum of saturated FAs (C12:0 to C22:0)

^3^ MUFA = sum of monounsaturated FAs (C16:1 to C22:1)

^4^ PUFA = sum of polyunsaturated FAs (C18:2 to C22:6)

^5^ UFA = sum of unsaturated FAs (MUFA + PUFA).

A large number of factors play a part in determining the fatty acid composition of muscles of common carp [32,33]. However, as environmental factors were controlled for in this experiment, the differences observed should be largely attributable to genetics [33]. The observed reduction in the level of oleic acid (MUFA: C18:1) is not a desirable result, as this FA is beneficial both for the total cholesterol and low-density lipoprotein cholesterol levels in plasma in humans and for the fish meat flavour [34]. However, the lower proportion of this MUFA in the Selected group is compensated by the increase in essential PUFAs, the consumption of which is associated with numerous health benefits [11,13]. Therefore, we can conclude that selection for faster growth did not negatively affect the meat quality (at least not in terms of FA composition) of the Selected line.

### Transcriptome analyses

The sequencing of twelve intestine samples, which includes eight specimens from the Selected group and four specimens from the Control group, yielded 93.78 Gb of clean data, reaching 6.17 Gb for a single sample, with Q30 over 87% (S1 Table). Clean reads of each sample were compared to the designated common carp reference genome, with mapping successfulness rates ranging from 62.97% to 64.91%. On the basis of the results of alternative splicing prediction, gene structure optimization, and queries of seven different databases, 5 346 new genes were discovered. Differentially expressed genes (DEGs) were identified by statistically comparing the results between the two groups. Functional annotation and enrichment analysis of 106 discovered DEGs was carried out subsequently. Data are deposited in the NCBI’s BioProject (PRJNA414702) and SRA (SRR6202422, SRR6202423, SRR6202418, SRR6202419, SRR6202424, SRR6202425, SRR6202421, SRR6202426, SRR6202420, SRR6202429, SRR6202427, SRR6202428) databases.

**Fig 1:**
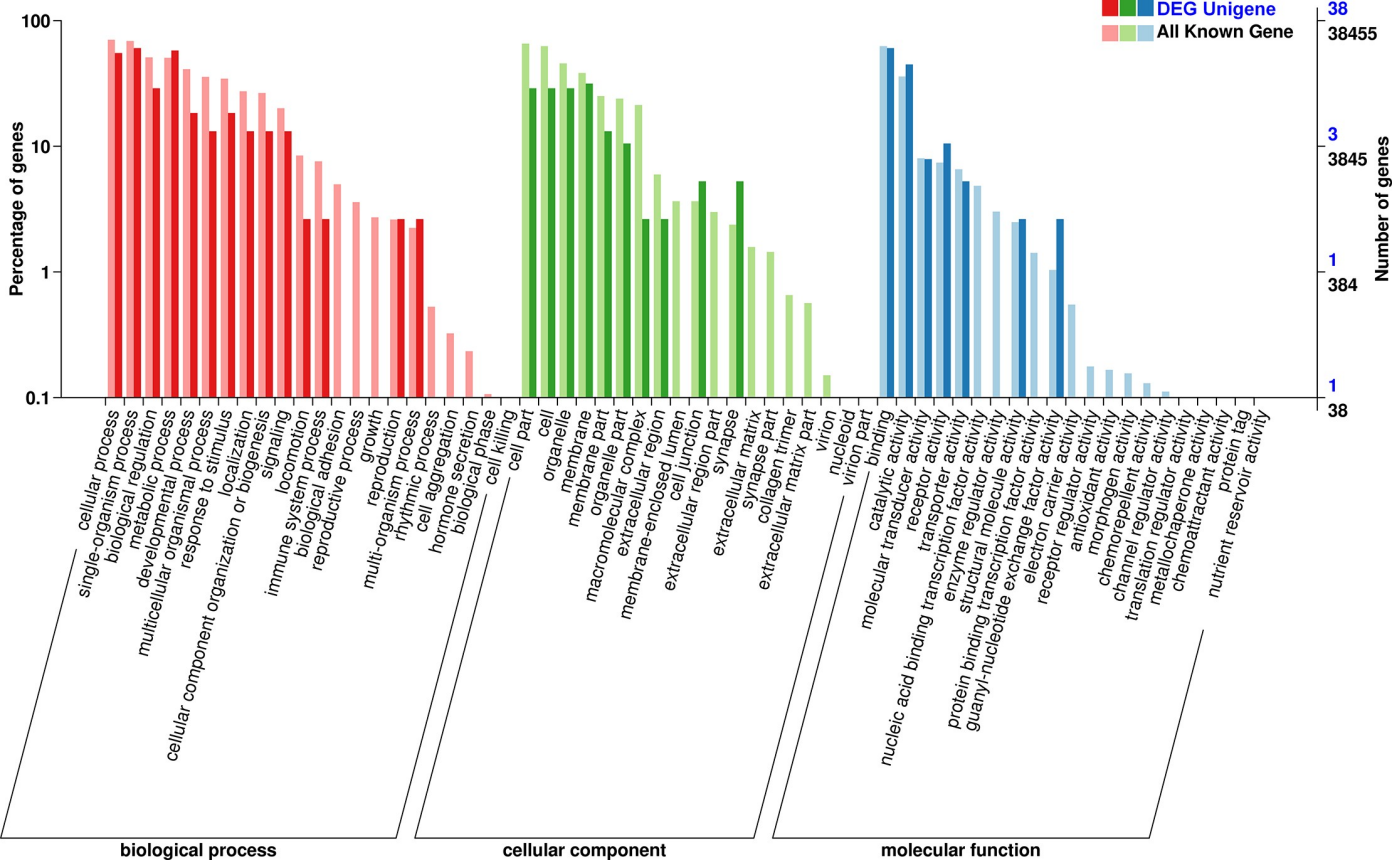
GO functional classification of annotated genes in transcriptome profiles of selection and control groups. ‘DEG Unigene’ refers to differentially expressed genes and ‘All Known Gene’ to all annotated genes in both groups.

Overall distribution of gene ontology terms following the GO database [35] annotation results are comparable to those previously reported for liver samples of bighead carp [23], with the exception of a strong increase in the percentage of DEGs associated with synapse (cellular component) in the intestinal samples of common carp. Comparison of functional category distribution between DEGs and the background transcriptome revealed notable differences in the percentages of genes associated with the following biological processes: biological adhesion, reproductive process and growth, all of which represented between 4 and 8% of all annotated genes, but were completely absent from the DEGs. In the cellular components category, notable differences were observed in the percentage of genes associated with macromolecular complexes, membrane-enclosed lumen, extracellular region part, extracellular matrix and synapse part, all of which appear to be notably less abundant, or even completely absent, from the DEGs. In the molecular function category, notable differences were observed in the percentages of genes associated with nucleic acid binding transcription factor activity and enzyme regulator activity, both of which represented between 3 and 7% of all annotated genes, but were completely absent from the DEGs. Among the categories with higher proportion of DEGs than all annotated genes were (in approximately descending order): guanyl-nucleotyde exchange factor activity (1% among all genes and ≈ 5% among DEGs), synapse, cell junction, receptor activity, metabolic process, multi-organism process, catalytic activity, and structural molecule activity (Fig 1).

**Fig 2:**
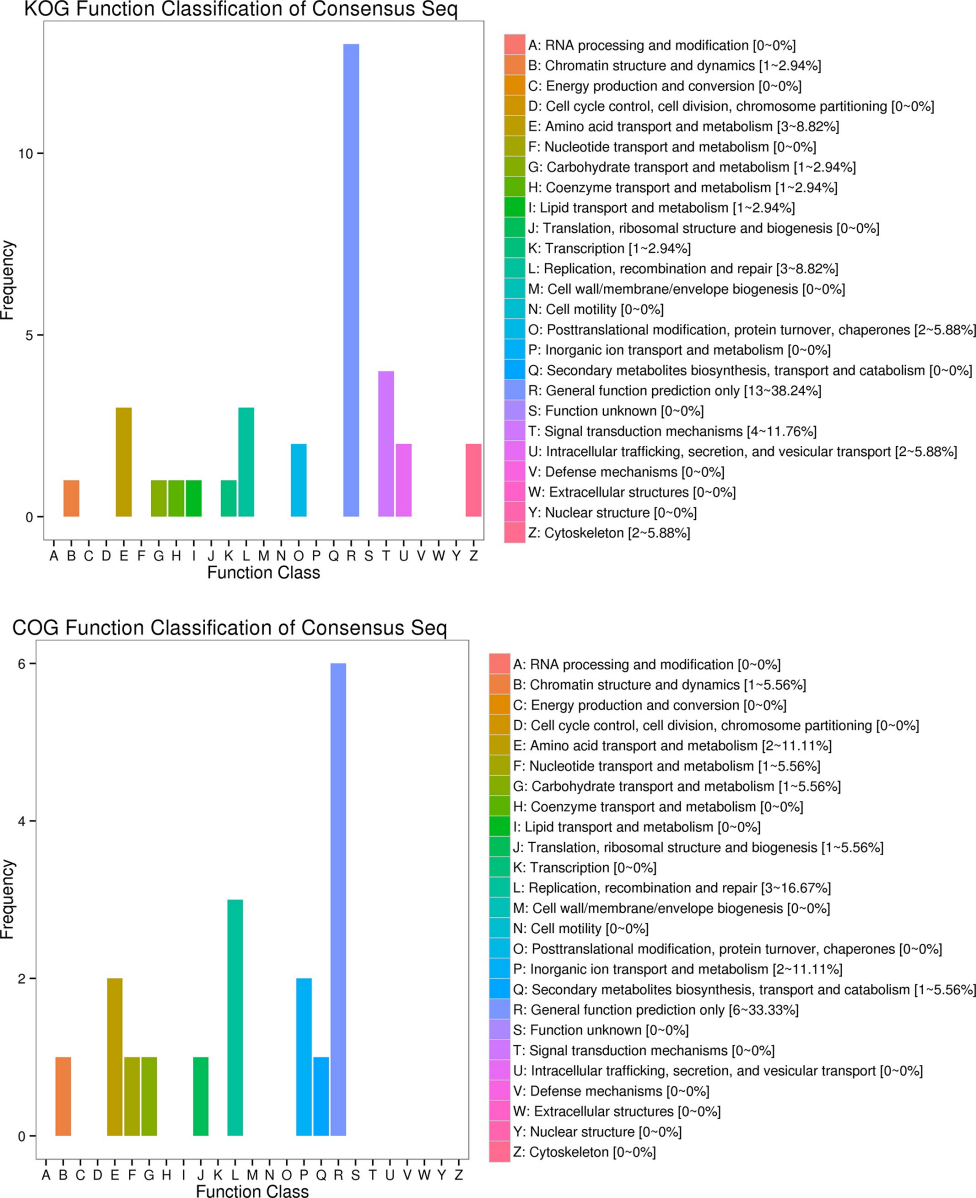
KOG and COG functional classification of differentially expressed genes between the selection and control groups.

Functions of DEGs were also predicted and classified by searching COG [36] and KOG [37] databases (Fig 2). Results of these two functional classifications were only partially congruent: by far the highest proportion of DEGs could be merely assigned to the ‘general function prediction only’ functional class in both classifications. In KOG classification, this was followed by ‘signal transduction mechanisms’, which was completely absent from COG, but ‘replication, recombination and repair’ and ‘amino acid transport and metabolism’ were found in both classifications. A small proportion of DEGs were associated with categories likely to be implicated in growth, such as carbohydrate and lipid (absent from COG) transport and metabolism. Intriguingly, although it may be expected that faster growth would be reflected in a number of DEGs related to the energy production, cell growth and division, DEGs classified as ‘energy production and conversion’, ‘cell cycle control, cell division, chromosome partitioning’, ‘cell wall biogenesis’, and ‘extracellular structures’ were absent from both classifications. This scarcity of functional categories of DEGs becomes even more obvious when the results are compared with liver profiles of fast and slow-growing bighead carp, where all functional categories were represented among the discovered DEGs [23]. This is almost certainly at least partially a reflection of the central regulatory role the liver has in the growth of fish [7], as opposed to very specific functions of intestines. However, importantly for the objectives of our study, pathway enrichment analysis showed that (only) endocytosis pathway was significantly enriched in DEGs, compared to the whole genome background (S1 Fig). This finding indicates that endocytosis efficiency might indeed contribute to the observed differences in the growth rates of two groups.

## Specific differentially regulated genes

**Fig 3:**
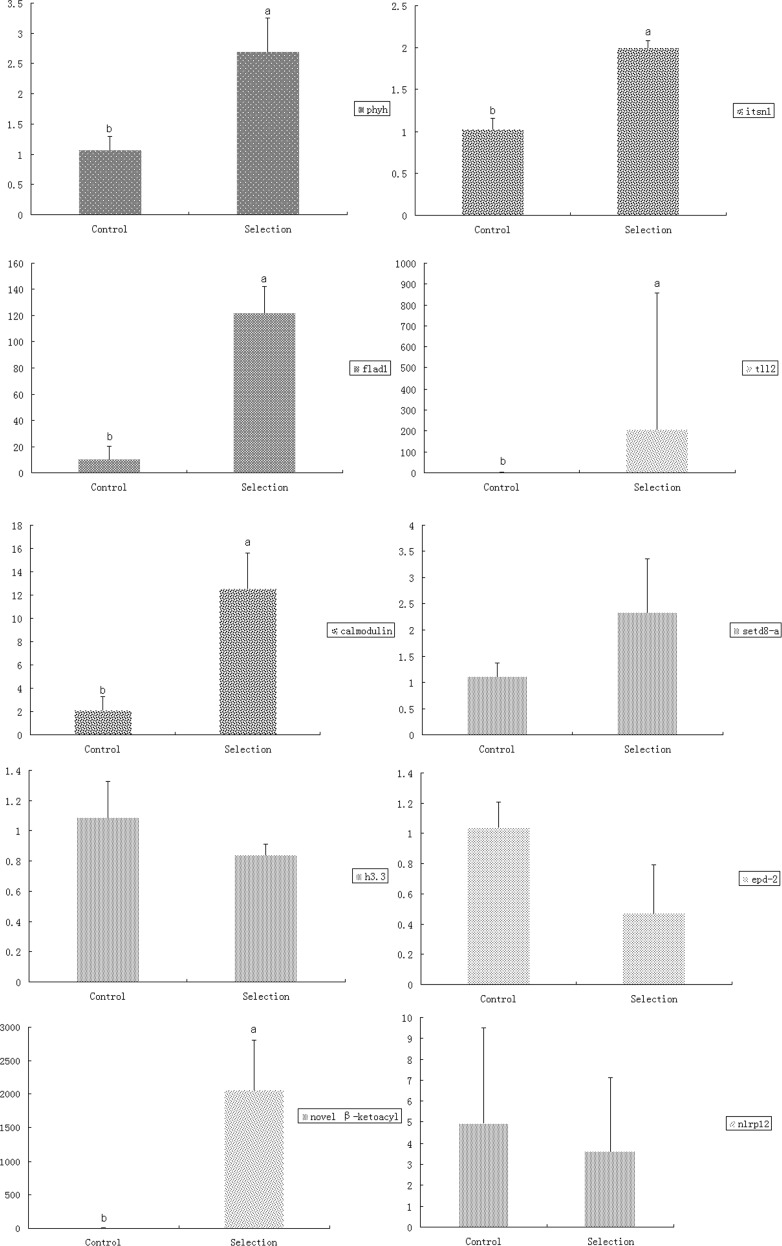
qPCR analysis of a subset of ten differentially expressed genes. Data were normalized to *gapdh* as the reference gene and presented as a fold change between the Selected and Control groups (mean±SD). Results were analyzed by the t-test, where different letters indicate significant (*P* < 0.05) differences.

Among the 106 DEGs (S2 Table), we have selected a subset of 25 genes which might be particularly relevant for the objectives of this study (Table 2). Overall, among the DEGs, there were more downregulated than upregulated genes. To corroborate the results of the transcriptome analysis, the expression of a further subset of ten of these DEGs was studied using qPCR (Fig 3). Correlation between transcriptomic and qPCR results was relatively low (S2 Fig), but this was mostly caused by a number of outliers, whereas some genes exhibited almost perfectly congruent results between the two methods (S3 Table). As discussed before [38], this might be a consequence of different data normalization methods used for qPCR (reference gene) and RNA-seq (RPKM) data analyses [39,40]. Gene expression patterns vary widely between tissues in common carp, which poses a problem for RNA-Seq analyses, which assume that samples are comparable [27,39]. These high individual gene expression differences may also explain the relatively low number of DEGs observed, as a large SD would influence the statistical significance calculations. Furthermore, gene expression analyses in teleost fishes are additionally encumbered by a large number of co-expressed, highly similar paralogs [38]: as a result of a relatively recent (≈8 MYA) genome duplication specific to this taxonomic group, teleost fishes possess a large number of duplicated genes [41]. Although many of the superfluous gene copies have been lost in the course of evolution, in many cases two (or more) paralogs have retained their functions and continue to be expressed [42,43]. In common carp, which is an allotetraploid, possessing a large number of functional paralogs [27,44,45], this can be up to four functional paralogs. As observed in our transcriptomic results as well, sometimes paralogs forming teleost gene families are differentially expressed, which indicates that this functional redundancy may have resulted in the evolution of slightly diversified functions and very complex regulation [5,46,47]. Apart from presenting a problem for gene annotation, these paralogs can also produce apparently inconsistent expression results. For example, in cases where qPCR analysis indicates upregulation, whereas the transcriptomics indicates downregulation, it is possible that the primers designed for qPCR may have non-specifically amplified both (or more) differentially expressed paralogs present in the genome.

**Table 2:** A subset of 25 putatively relevant genes among those differentially expressed in Selected and Control groups.

Gene Name	Nr or Swiss Prot^2^ annotation	eggNOG class annotation	FDR	log2FC	Reg
*phyh*	Phytanoyl-CoA dioxygenase, peroxisomal precursor [Osmerus mordax]	Lipid transport and metabolism	0.003956	2.31	up
*il2rb*	Interleukin-2 receptor subunit beta precursor [Danio rerio]	Extracellular structures	0.000423	Inf	up
*H3*.*3*	Histone H3.3 [Salmo salar]	Chromatin structure and dynamics	7.54E-07	Inf	up
*itsn1*	Intersectin or EH-domain containing 1a [D. rerio]	Signal transduction^1^	0.003146	1.63	up
*novel β-ketoacyl*	Novel protein containing a beta-ketoacyl synthase, N-terminal domain [D. rerio]	Lipid transport and metabolism	0.002079	11.54	up
*flad1*	FAD synthase region [D. rerio]^2^	Coenzyme transport and metabolism	2.05E-17	5.77	up
*tll2*	PRED: cubilin [D. rerio]; Tolloid-like protein 2 [Xenopus laevis ]^2^	Coenzyme transport and metabolism	0.00392	Inf	up
*cd276*	CD276 antigen homolog (Precursor) [X. laevis ]^2^	General function prediction only	3.27E-07	Inf	up
*calm*	Calmodulin [Oreochromis mossambicus] ^2^	Signal transduction mechanisms	0.004969	7.96	up
*nlrp3-like*	PRED: NACHT, LRR and PYD domains-containing protein 3-like [D. rerio]^3^	General function prediction only	1.16E-05	2.43	up
*nlrp3*	PRED: NACHT, LRR and PYD domains-containing protein 3^5^ [D. rerio]	General function prediction only	^5^	^5^	down
*nlrp12*	PRED: NACHT, LRR and PYD domains-containing protein 12^5^ [D. rerio]	General function prediction only	^5^	^5^	down
*setd8-a*	PRED: N-lysine methyltransferase SETD8-like [D. rerio]	General function prediction only	0.000502	-5.01	down
*rpgrip1l*	RPGRIP1-like (K16550)^6^	Embryonic pattern specification^7^	0.000491	-inf	down
*atp10-b*	PRED: probable phospholipid-transporting ATPase VB [D. rerio]	Inorganic ion transport and metabolism	2.63E-06	-inf	down
*epd-2*	PRED: ependymin-2-like [Astyanax mexicanus]	General function prediction only	0.003711	-1.93	down
*ldlrad3*	PRED: low-density lipoprotein receptor class A domain-containing protein 4-like [D. rerio]	Signal transduction mechanisms	0.001095	-3.99	down
*glomulin*	PRED: glomulin, FKBP associated protein b isoform X1 [D. rerio]	Function unknown	5.38E-14	-6.16	down
*aggf1*	Angiogenic factor with G patch and FHA domains 1 [D. rerio]	General function prediction only	0.00088	-1.73	down
*cxcl14*	Cxcl14 protein [D. rerio]	General function prediction only	0.000458	-1.49	down
*tln2*	PRED: talin-2 isoform X2 [D. rerio]	Cytoskeleton	0.00066	-1.50	down
*svop*	PRED: synaptic vesicle glycoprotein 2C-like [A. mexicanus]	General function prediction only	1.43E-05	-5.91	down
*st8sia1*	alpha 2,8-sialyltransferase ST8Sia I/V/VI-r2 [D. rerio]	Carbohydrate transport and metabolism	0.000165	-6.39	down
*proteoglycan 4-*like	PRED: proteoglycan 4-like, partial [Poecilia reticulata]	-	8.88E-05	-inf	down
*trim25*	PRED: E3 ubiquitin/ISG15 ligase TRIM25 isoform X3 [D. rerio]^4^	Posttranslational modification, protein turnover, chaperones	1.60E-07	-inf	down

PRED = predicted

^1^ KOG class annotation

^2^ Swiss Prot annotation

^3^ SwissProt annotation is Neoverrucotoxin subunit alpha [*Synanceia verrucosa*, Reef stonefish]

^4^ Annotation of this protein was ambiguous, depending on the database: E3 ubiquitin-protein ligase ORTHRUS 2-like in KEGG annotation, Stonustoxin subunit alpha [*Synanceia horrida*, Estuarine stonefish] in SwissProt

^5^ Eight isoforms of this gene were downregulated, five of which were annotated as protein 3(-like) and three were protein 12(-like). FDR values ranged from 1.17E-07 to 5.16E-03 (2.08E-03 on average) for protein 12-like, and from 8.92E-07 to 0.000787 (0.000251 on average) for protein 3-like. Regulation ranged from -2.02 to -2.31-fold (-2.17 on average) for protein 12-like, and from -2.01 to -5.74-fold (-3.61 on average) for protein 3-like. Two of the isoforms (one protein-3 and one protein-12) were annotated as Protein NLRC5 (gene name = nlrc5) [*Ictalurus punctatus*, Channel catfish] in SwissProt database

^6^ KEGG annotation

^7^ GO annotation.

A number of regulated genes could be associated with metabolism, growth, development and proliferation. Most importantly for this study, two upregulated genes were associated with endocytosis pathway: *interleukin-2 receptor subunit beta* (*il2rb*; infinite upregulation) and *EH domain-containing protein 1* (*ehd1* or *intersectin-1*; +1.6). The *il2rb* gene is involved in receptor-mediated endocytosis and transduces the mitogenic signals of *il2* [48]. This gene has not been associated with animal growth before (to our knowledge), but a very recent study indicated that it might be implicated in jaw diversity in pupfishes [49]. The Ehd1 protein interacts with Insulin-like growth factor 1 (Igf-1) receptor. The level of Igf-1 in serum is known to be positively correlated with growth rate in fishes [3]. Generally, in most vertebrates, growth hormone/insulin-like growth factor (Gh/Igf) axis (a major component of which is Igf-1) plays a key role in the regulation of growth, proliferation and differentiation [6,7,9,21,50]. Particularly interesting for this study is that Gh supports muscle protein synthesis indirectly by enhancing rates of amino acid uptake from the intestine and by stimulating intestinal growth [3,51]. This indicates that the higher growth rate observed in the Selected group is likely to be at least partially attributed to increased endocytosis efficiency.

Two of the upregulated genes are known to be involved in the fatty acid metabolism: the enzyme encoded by *phytanoyl-CoA dioxygenase*, *peroxisomal* (*phyh*) gene (transcriptome = 2.3, qPCr = 2.5) is critical for the normal function of peroxisomes, which play a key role in the breakdown of some fatty acids [48,52]. A novel gene was annotated on the basis of its similarity with zebrafish genes: protein containing a beta-ketoacyl synthase, N-terminal domain. This gene, very highly upregulated in both transcriptome (11.5) and qPCR (710.79) analyses, is likely to be responsible for the chain-elongation step of dissociated (type II) fatty-acid biosynthesis, i.e. the addition of two C atoms to the fatty-acid chain [53].

Several other genes upregulated in the Selected group are likely to be indicators of heightened transcription levels: *histone h3*.*3* gene was extremely highly upregulated in the transcriptome analysis (infinite), but only mildly upregulated (0.77) in the qPCR analysis. This gene is believed to represent an epigenetic imprint of transcriptionally active chromatin [48]. Three more upregulated genes are associated with transcription and RNA processing [54]: *rna-binding protein 5* (2.01), *n-lysine methyltransferase setd8-a* (4.15) and *fad synthase* region (*flad1*), which was very highly upregulated in both transcriptome (5.77) and qPCR (11.94) analyses. Intriguingly, two *setd8-a* paralogs were downregulated in the transcriptomic analysis (-5 and -infinite), which indicates that *setd8* paralogs might have slightly different functions and complex regulation.

Several more DEGs are associated with metabolic and developmental roles: *tolloid-like protein 2* (*tll2*) gene encodes a protease which processes procollagen C-propeptides, such as chordin. It is required for the embryonic development, where it influences dorso-ventral patterning and skeletogenesis [48,55,56]. It appears to have a broad range of functions, as it has been linked with behaviour in mice and bipolar disorder in humans [57]. Intriguingly, a knockout of this gene resulted in increased muscle mass in mice [58], which is not in agreement with our observations (positive correlation). This gene was extremely highly upregulated in both analyses: infinitely in transcriptome and 610.70 in qPCR. As tolloids were also associated with gut regeneration in sea cucumber; the authors proposed that Bmp1/Tll axis might be involved in folding of the luminal epithelium and gut looping [59]. This raises some interesting hypotheses about the correlation between *tll2* expression, proliferation of intestinal epithelium, and growth in common carp, but they need to be further experimentally tested. *Calmodulin* was very highly upregulated in both analyses: 7.96 in transcriptome and 5.92 in qPCR. It mediates the control of a large number of enzymes, including protein kinases and phosphatases [48]. Calmodulin plays an important role in the activation of phosphorylase kinase, which ultimately leads to glucose being cleaved from glycogen by glycogen phosphorylase. It also plays an important role in lipid metabolism by activating calcitonin, which is a hormone that lowers blood Ca^2+^ levels and activates G Protein cascades that lead to the generation of cAMP [48]. In fish, calmodulin has been proposed as a molecular stress indicator [60]. *Phospholipid-transporting ATPase IC* gene (*atp10b*), a component of the P4-ATPase flippase complex, which catalyzes the hydrolysis of ATP coupled to the transport of aminophospholipids from the outer to the inner leaflet of various membranes and ensures the maintenance of asymmetric distribution of phospholipids [48], was strongly downregulated (- infinite). Phospholipid translocation also seems to be implicated in vesicle formation and in uptake of lipid signaling molecules [48]. The protein is found in brain and in low levels in testis, but its expression is known to be enhanced in intestines in humans [61,62]. However, the reason for its strong downregulation in the Selected group remains unclear.

Intriguingly, two downregulated genes are associated with vision in animals: *rpgrip1-like* gene (- infinite) encodes a photoreceptor protein that is a key component of cone and rod photoreceptor cells. Mutations in this gene lead to autosomal recessive congenital blindness [54,63,64]. *Ependymin-2-like* (*epd*) was downloaded in the transcriptome analysis (-1.9) and slightly upregulated (0.46) in the qPCR analysis. It belongs to the family of proteins predominant in the cerebrospinal fluid of teleost fishes, associated with neuroplasticity and (optic nerve) regeneration [65]. This particular gene (*epd-2*), however, is expressed in non-brain tissues in fishes. In intestines, it is overexpressed in colon cancer, and its overexpression might be associated with intestinal regeneration [65]. It has also been associated with aggression levels in teleosts: inactivation of *ependymin* in subdominant fish resulted in a substantial increase in aggression in parallel with an enhanced competitive ability [66]. Therefore, lower expression of this gene in the Selected group may result in more aggressive behaviour during feeding [67] and ultimately faster growth of the fish in this group. Two more downregulated genes have been associated with neuronal function: *ST8 Alpha-N-Acetyl-Neuraminide Alpha-2*,*8-Sialyltransferase 1* (*st8sia1*: -6.4) has been associated with multiple sclerosis, as well as with metabolism of membrane-bound sphingolipids, important for cell adhesion and growth of cultured malignant cells [68]. *Synaptic vesicle 2-related* (*svop*; -5.9) is involved in neuron formation, maturation, or neuronal function [69]. Another gene associated with multiple functions in the synaptic vesicle cycle [70], *itsn1*, was upregulated in both transcriptome (1.63) and qPCR (1.94) analyses. *Low-density lipoprotein receptor class A domain-containing protein 3* (*ldlrad3*), an important component of a pathway associated with neurodegenerative diseases, including Alzheimer’s [54,71], was strongly downregulated (-4.0). Similarly, *rhoGEF and pleckstrin domain-containing protein 1* (*farp1*) gene, also relatively strongly downregulated (-2.7), plays a role in the assembly and disassembly of dendritic filopodia, the formation of dendritic spines, regulation of dendrite length and ultimately the formation of synapses [48]. It is not clear whether mRNAs of these genes originate from the enteric nervous system, or whether some of them are expressed in cells of the gastrointestinal wall. Regardless, these results indicate that genetically-driven behavioural differences (higher aggression levels) may also have contributed to the higher growth of the Selected group. However, there are also indications that this population might have a higher propensity for inherited diseases leading to sight impairment, as well higher propensity towards neurodegenerative diseases in general.

A number of regulated genes were associated with the immune system: *cd276* antigen homolog (upregulated: infinite) modulates immune responses, *trim25* gene (downregulated: -infinite) is involved in innate immune defense against viruses, and *cxcl14* (downregulated: -1.5) belongs to the cytokine gene family, which encode secreted proteins involved in immunoregulatory and inflammatory processes [48]. *cxcl14* might also be involved in the homeostasis of monocyte-derived macrophages, rather than in inflammation [54]. Nine gene isoforms belonging to the NACHT, LRR and PYD domains-containing protein (*nlrp*) family were also regulated: three *nlrp12* gene isoforms and six *nlrp3* gene isoforms. Apart from one *nlrp3* isoform (upregulated: 2.43), all other isoforms were downregulated: *nlrp3* from -2.01 to -5.74, and *nlrp12* from -2.02 to -2.31. Whereas in mammals this family contains only several members, in teleost fish, NLR proteins have expanded into a huge family containing hundreds of genes, which mostly act as innate immune sensors for pathogen-associated stress signals [72]. Therefore, if we presume that studied carps were exposed to similar pathogens, downregulation of these paralogs indicates a possibility of slightly impaired immune responses of the Selected group.

Two downregulated genes are associated with the development of vasculature: mutations in *glomulin* (-6.2) have been associated with glomuvenous malformations [73], whereas *angiogenic factor with G patch and FHA domains 1 g*ene (*aggf1*; -1.73) promotes angiogenesis and proliferation of endothelial cells. Another two downregulated genes might be an indication of a higher likelihood of health-related problems in the Selected group: *talin-1* (*tln1*; -1.5) is involved in connections of major cytoskeletal structures to the plasma membrane and cell-cell contacts [48], whereas *proteoglycan 4-like* (-infinite) prevents protein deposition onto cartilage from synovial fluid by controlling adhesion-dependent synovial growth and inhibiting the adhesion of synovial cells to the cartilage surface [48].

To sum up the results: two upregulated genes are known to be involved in the fatty acid metabolism, several genes are likely to be indicators of heightened transcription levels and proliferation, several are associated with metabolic and developmental roles, several with neuronal functions (including two with vision), several with the immune system, and two downregulated genes with the development of vasculature.

## Conclusions

Gene expression analyses indicate that the higher growth rate of the Selected group is likely to be at least partially attributed to increased endocytosis efficiency and genetically-driven behavioural differences (higher aggression levels). Among the detected DEGs, *il2rb* is the most likely candidate gene to explain the increased growth rate, while *phyh* is the most likely candidate to explain the increased unsaturated fatty acid content in the muscles of the new Huanghe carp strain. Our results contribute to the understanding of the genetic basis for growth and unsaturated fatty acid content in fish, as well as vertebrates in general.

## Materials and methods

### Strain creation

Following the national aquaculture development strategy, a new strain of common carp was created at the Nanquan farm of the Freshwater Fishery Research Center (FFRC) of Chinese Fisheries Academy in the period between 2010 and 2016 with the aim of achieving higher growth rate and unsaturated fatty acid muscle content, and lower feeding costs [31]. The basis for the strain creation was a population (n = 1600) of the ‘Yellow River’ (Yuxuan Huanghe) strain specimens introduced from the Fisheries Science Research Institute of the Henan Academy of Fishery Sciences and a local Jian carp population (n = 20). A subpopulation of about 600 Huanghe and 20 Jian specimens exhibiting a comparatively high growth rate was selected in 2010 for the creation of the new strain (age ≈ 17-month-old, average weight = 197.40±75.08 g) via artificial breeding (S3 Fig). The best model of parental pair selection was inferred via BLUP analysis [74] on the basis of their breeding values and inbreeding coefficients (<0.02). ‘Selected’ line was created in the following way: families and individuals in each family were ranked according to their breeding values, then male and female specimens with highest breeding values were selected from the top-ranking families, and their inbreeding coefficient values were calculated. If the inbreeding coefficient value of a given pair was below 0.02 it was chosen for the Selected line family. A total of approximately 60 Selected line families (the number varied among generations) were created in this way, with each family represented by 50 (offspring) specimens produced by a selected parental pair. The control line (average number of families per generation = 20) was generated by random mating of specimens with inbreeding coefficient < 0.02. Their breeding values and the best model for parental pair selection were inferred via a BLUP analysis. When they reached the size of about 10 g (≈ 3 months of age), F1 juveniles were PIT (Passive Integrated Transponder) tagged for subsequent identification, and growth performance data (length and weight) were simultaneously collected. At 8 months of age, growth performance of the F1 offspring was measured again, which was followed by a comprehensive screening for molecular markers, BLUP analysis, and unsaturated fatty acid muscle content analysis, to estimate the breeding value of the broodstock. For the molecular markers we selected five markers (SNPs and SSRs) which we previously found to be associated with body weight in carp: *IGF2a*^4#^, *IGF2R* intron 1, *D-LOOP*253, *COI*626, and Koi42 [75]. DNA samples of parents and selected (high-performing) 500 offspring were also collected. F2 parents were selected on the basis of juvenile growth performance and their molecular markers. The best model for parental pair selection was obtained via data integration with the help of SAS/STAT 8.0 (SAS Institute Inc., Cary, NC, USA) and R3.1.14 [76] programs. The breeding value was estimated using ASReml2 [77], DMU [78], Python [79], and two unpublished (but copyrighted) in-house developed programs, DNA compare1.0 and COECAL2.1. This process was repeated for three generations (2010–2016, two years per generation; S3 Fig): one-to-one artificial propagation of the core population was conducted in the early May of 2012, 2014 and 2016 following the standard procedure [31,80]. In 2016, after three generations of selection and breeding, we had a stock of 4000 (80 × 50) selected F3 specimens, which were released into the same pond for the breeding program (not further discussed in this study). In addition to these, we randomly selected five specimens from 48 families (5 × 48 = 240 specimens) for the growth performance test (cage culture experiment) and unsaturated fatty acid muscle content analyses. Among these 48 families, 38 were from the Selected line and 10 were from the control group. When they reached the size of about 10 g (≈ 3 months of age), all 240 juveniles were PIT (Passive Integrated Transponder) tagged for subsequent identification.

### Growth performance and tissue sampling

To test the growth performance of the new strain, the aforementioned 240 healthy one-year-old F3 generation specimens were reared in eight 1×1×1.2 m cages (approx. 30 fish per cage) placed in earthen ponds from early September to mid-November 2016 (8 weeks). Selected and Control lines were reared together, and there was no significant difference in the initial weight (at the onset of the cage-rearing experiment) between the eight groups (cages). Light/dark schedule was natural. Fish were fed pelleted feed (S4 Table) three times daily (8:00, 12:00, 16:00) to satiation (until fish would stop taking floating pellets from the surface). The amount of feed was, thus, variable. Hand clapping was used as the feeding signal. The last feeding was on the 56^th^ day, 8:00am, at which point the fish was eight months-old. After starving the fish for 24h, at 8:30am of the 57^th^ day all 240 individuals (mortality rate = 0.0) were tranquilised in 20–30 mg/L MS-222 (Sigma-Aldrich, USA) as described [81]. Selected morphometric traits were recorded: weight (g, 2 kg scale), standard length, body depth, body width (all to mm, using a ruler) (S5 Table). One family from both lines (Control and Selected) was randomly chosen for tissue sampling, followed by random selection of eight specimens from the Selected group and four specimens from the Control group (S1 and S5 Tables). These twelve specimens (5 male + 7 female, S1 Table) were euthanised in buffered MS-222 at 350 mg/L concentration [81]. The fish were then dissected and a caudal portion of the epaxial muscle (> 10 g) sampled using sterile scissors and forceps, and placed in sterile plastic bags. After weighing the entire viscera, the intestine was rinsed three times with sterile phosphate buffered saline (PBS, pH = 7.0) to remove the ingesta, and foregut, midgut and hindgut separated as described [82]. Samples were immediately (<3min) placed in labelled sterile plastic bags, flash-frozen in liquid nitrogen, and stored at -80°C. The handling of animals was conducted in accordance with the guidelines for the care and use of animals for scientific purposes set by the Institutional Animal Care and Use Committee of the Freshwater Fisheries Research Center, Chinese Academy of Fishery Sciences, Wuxi, China and the EU Directive 2010/63/EU for animal experiments. The permit to conduct this study was obtained from the institutional Animal Care and Use Committee of the Freshwater Fisheries Research Center, Chinese Academy of Fishery Sciences.

### Analysis of fatty acid composition

To determine the effects of selection for fast growth on fatty acid (FA) composition of carp meat, muscle samples of eight specimens from the Selected group and four specimens from the Control group were collected (described above) and analysed. As fish muscles are mostly composed of long-chain fatty acids [33,83], we focused on the composition of long-chain (C12 to C22) FAs in this experiment. A portion (≈4 g) of each muscle sample was manually crushed in liquid nitrogen using mortar and pestle and sent (on ice) to the State Key Laboratory of Food Science (Jiangnan University, Beijing), where the total lipid extraction and analysis was performed as described before [33,84]. Briefly, lipids were extracted using chloroform/methanol (v/v = 2:1), methylated with 10% (v/v) methanolic HCl at 60°C for 3 h, extracted with n-hexane, and analysed by gas chromatography using Agilent DB-WAXETR column (Agilent Technologies, Santa Clara, CA, US), according to the manufacturer’s protocol. Pentadecanoic acid (15:0) was used as the internal standard. Statistical analysis was conducted in Excel, with the significance threshold set at 0.05.

### Transcriptome analysis

#### RNA isolation, cDNA library construction and Illumina sequencing

Total RNA for each sample was extracted from the intestine samples, cDNA libraries constructed and sequenced, and transcriptome assembled and annotated by the Beijing Biotech Co. Ltd. (Beijing, China) roughly as described before [85,86]. In total, eight specimens from the Selected group and four specimens from the Control group were used for the transcriptome analyses. To ensure that foregut, midgut and hindgut segments are represented in the transcriptome, RNA was extracted (Trizol, Invitrogen, US) separately from the three segments, and equal amounts (of the three segments belonging to the same specimen) were then pooled together. RNA integrity and concentration were assessed using an Agilent Bioanalyzer 2100 system (Agilent Technologies, CA, USA). cDNA libraries were generated using NEBNext UltraTM RNA Library Prep Kit for Illumina (NEB, USA) following the manufacturer’s recommendations. One μg of RNA per sample was used as input material. Index codes were added to attribute sequences to each sample. Briefly: poly-T oligo-attached magnetic beads (NEB, E7490) were used to purify the mRNA from total RNA. Fragmentation was carried out using divalent cations under elevated temperature in NEBNext First Strand Synthesis Reaction Buffer (5X). First strand cDNA was synthesized using random hexamer primer and M-MuLV Reverse Transcriptase (RNase H). Second strand cDNA synthesis was subsequently performed using DNA Polymerase I and RNase H. Remaining overhangs were converted into blunt ends via exonuclease/polymerase activities. After adenylation of 3’ ends of DNA fragments, NEBNext adaptors with hairpin loop structure were ligated to prepare for hybridisation. In order to select cDNA fragments of preferentially 200–250 bp in length, library fragments were purified with AMPure XP system (Beckman Coulter, Beverly, MA, USA). Following this, 3 μl of USER Enzyme (NEB, USA) was incubated with size-selected, adaptor-ligated cDNA at 37°C for 15 min, followed by 5 min at 95°C. PCR was then performed with Phusion High-Fidelity DNA polymerase, universal PCR primers and Index (X) Primer. Finally, PCR products were purified (AMPure XP system) and library quality assessed on the Agilent Bioanalyzer 2100 system. Clustering of the index-coded samples was performed on a cBot Cluster Generation System using TruSeq PE Cluster Kit v4-cBot-HS (Illumina) according to the manufacturer’s instructions. After cluster generation, the library preparations were sequenced on an Illumina Hiseq 2500 platform and paired-end reads were generated.

#### Transcriptome assembly, annotation, ontology, and differential gene expression

Raw data (raw reads) were first processed through in-house perl scripts WipeAadpter.pl and Fastq_filter (Biomarker Technologies, Beijing): adaptor-only reads, reads containing poly-N stretches (>5% total N), and low-quality reads (Q20-value≤20) were filtered out, thereby producing ‘clean reads’. These clean reads of each sample were compared to the designated reference common carp genome downloaded from the CarpBase [87] using TopHat2 [88], based on Bowtie2 algorithm [89]. The numbers of reads produced by RNA-Seq analyses were normalized to FPKM (fragments per kilobase of transcripts per million fragments mapped) to compute gene expression levels [90], and differentially expressed genes (DEGs) between the two groups (S and C) detected using EBseq software [91]. Benjamini-Hochberg procedure [92] was used to control the false discovery rate (FDR). Genes were defined as differentially expressed when they exhibited the following parameters: |fold change|≥ 2 and FDR < 0.01. Genes were queried against a number of databases using BLASTx [93]: RefSeq [94], UniProt [95], GO [35], COG [36], KOG [37], Pfam [96], and KEGG [97]. After using KOBAS 2.0 [98] to obtain the KEGG Orthology results, HMMER [99] web server was employed to obtain the annotation for new genes from the predicted amino acid sequences using Pfam database. Pathway enrichment analysis was conducted using KEGG database as described before [100].

### qPCR

This method was used to further investigate the expression of a subset of ten genes significantly differentially expressed between the two groups (eight specimens for the Selected group and four specimens for the Control group). Genes were selected based on their relevance for the objectives of this study and regulation magnitude. Total RNA was extracted prepared as described above from the same samples that were used for the transcriptome sequencing. cDNA libraries were prepared using PrimeScriptTM RT reagent Kit with gDNA Eraser (Takara) following the manufacturer’s protocol. cDNA libraries were diluted five-fold and used as templates for qPCR with the primers listed in Table 3. Three reference genes, *gapdh*, *beta actin* and *18S*, were tested for the stability of expression among all samples; as reflected in no significant differences between Selection and Control groups (S6 Table), all three genes exhibited a stable expression. On the basis of our previous experiences and published studies [101,102], we selected *gapdh* as the reference gene. Primers were designed using Primer Premier 5 software (Premier Biosoft, USA) on the basis of sequences of these ten genes obtained from the transcriptome data, and synthesized by the WCcgene company (Shanghai, China). qPCR was performed using ABI VIIA@7 instrument (ABI, USA) and GoTaq qPCR Master Mix (Promega, USA). In brief: the total qPCR mixture reaction volume of 10 μL contained 5 μL GoTaq qPCR Master Mix 2X, 3 μL ddH_2_O, 0.75 μL of each primer and 0.5 μL of cDNA template. qPCR procedure: pre-incubation at 95°C for 10 min, followed by 40 cycles of 15 s at 95°C, 30 s at 60°C, and 30 s at 72°C. Melt curve analysis was performed at the end. Reactions were performed in triplicate for each sample. *gapdh* was chosen as the reference gene according to earlier recommendations [103,104]. Expression levels are presented as fold changes relative to the target gene expression in the control group, calculated using the 2^–ΔΔCt^ method [105]. qPCR data were analyzed statistically using Microsoft Excel and SPSS. Student-T test, implemented in SPSS, was applied to test the statistical significance of differences between the two groups, where significance thresholds were set at P < 0.05 (significant) and P < 0.01 (highly significant).

**Table 3:** Primers used for qPCR.

Gene	Primer sequence (5`-3`)	T_m_ (°C)	Fragment length
*phyh*	F: 5`-GCTCGTTGACTCTGTGCTGG-3`	60	400
	R: 5`- TCGTAATGCTTCGGCTTGTG-3`		
*itsn1*	F: 5`-GGCAGATGTTGACAAAGACG-3`	59	118
	R: 5`- CACAAGGTGAGCAGGCAGT-3`		
*flad1*	F: 5`-GCACCAAATCCTCCGACAG-3`	59	142
	R: 5`- TACCAGCAGAACAGCAACAA-3`C		
*tll2*	F: 5`-TCCAAAGGAGGATTTACTG-3`	52	179
	R: 5`- GATTTGATGGTGCCTGTAT-3`		
*calmodulin*	F: 5`-AGACAACTACCTGAGCACCTC-3`	57	130
	R: 5`- GACAAATAGCAGCCATCCA-3`		
*setd8-a*	F: 5`-ACAAGAGGCAGAAGAAGTA-3`	52	145
	R: 5`- TGCTAAAGTAGGAGGAAAC-3`		
*h3*.*3*	F: 5`-GCGAGTGCTGTTTGTTCTG-3`	57	95
	R: 5`- CTCGGTGGACTTCTGGTAG-3`		
*epd-2*	F: 5`-GTCTCGTCGCAGGGTGTAAT-3`	59	135
	R: 5`- CCAGACCAGGAACGGACAT-3`		
novel *β-ketoacyl*	F: 5`-TTCCCTACCCACGATACCA-3`	58	123
	R: 5`- ACATTGAACTGCCTGTGCC-3`		
*nlrp12*	F: 5`-TGACCAACAGAAGGAGCAG-3`	57	226
	R: 5`- CTGTATCCGCAGGAAGTGA-3`		
*18S*^*1*^	F: 5`-TTCCCTACCCACGATACCA-3`	60	
	R: 5`- ACATTGAACTGCCTGTGCC-3`		
*beta actin*^*1*^	F: 5`-TGACCAACAGAAGGAGCAG-3`	60	
	R: 5`- CTGTATCCGCAGGAAGTGA-3`		
*gapdh*^*1*^	F: 5`-CCGTTCATGCTATCACAGCTACACA-3`	62	310
	R: 5`- GTGGATACCACCTGGTCCTCTG-3`		

^1^ reference genes.

## Supporting information

S1 Table Transcriptome sequencing statistics for the 12 samples. (DOCX)

S2 Table Details of all DEGs. (XLSX)

S3 Table Comparison of gene expression results between transcriptomic and qPCR data. Log2FC is the result of transcriptome analysis, Reg is regulation, and inf is infinite.
(DOCX)

S4 Table Diet composition. (DOCX)

S5 Table Identification details and growth parameters for the 12 specimens selected for RNA-seq and fatty acid composition analyses. BWe = body weight (g), TL = total length (mm), CF = condition factor, BWi = body width (mm), BH = body height (mm). The last row in each group contains the average values of morphometric characteristics.
(DOCX)

S6 Table Ct values of three tested reference genes in all samples. (DOCX)

S1 Fig Endocytosis pathway enrichment analysis in the KEGG database. Significantly enriched genes are highlighted in red.
(PNG)

S2 Fig Correlation between transcriptome and qPCR data. Transcriptome results are presented on the x-axis (log_2_FC) and qPCR on the y-axis. The following outliers were removed from the analysis: *h3*.*3* and *tll2* (inf) and novel *β-ketoacyl* (>700) (see S3 Table).
(PDF)

S3 Fig New Huanghe carp strain selection workflow. (PDF)
