# Supplementary material for: Comparative transcriptomics identifies genes differentially expressed in the intestine of a new fast-growing strain of common carp with higher unsaturated fatty acid content in muscle
Source: PLoS One. 2018 Nov 5;13(11):e0206615. doi: 10.1371/journal.pone.0206615 (PMC6218049; doi:10.1371/journal.pone.0206615)
Supplement: S1 Table — (DOCX) [file pone.0206615.s001.docx]

**S1 Table. The numbers of clean reads, clean bases, GC content and Q30 values of the 12 specimens selected for RNA-seq analyses.**

| **Sample** | **Clean reads** | **Clean bases** | **GC Content** | **%≥Q30** |
| --- | --- | --- | --- | --- |
| T01 | 28531206 | 8505226776 | 46.60% | 87.43% |
| T02 | 29621604 | 8804937506 | 47.04% | 87.84% |
| T03 | 27092285 | 8057912926 | 48.21% | 87.17% |
| T04 | 29189022 | 8725862812 | 47.76% | 87.59% |
| T05 | 29615359 | 8802486844 | 47.33% | 87.60% |
| T06 | 28899130 | 8588918416 | 47.01% | 87.69% |
| T07 | 31484501 | 9402221882 | 46.59% | 86.86% |
| T08 | 25754288 | 7667266702 | 47.20% | 87.25% |
| T09 | 20790708 | 6165066888 | 46.77% | 87.28% |
| T10 | 21217716 | 6298810430 | 47.08% | 87.22% |
| T11 | 21829356 | 6526851906 | 47.81% | 87.10% |
| T12 | 20932271 | 6236552356 | 47.75% | 87.52% |
