# Supplementary material for: Comparative transcriptomics identifies genes differentially expressed in the intestine of a new fast-growing strain of common carp with higher unsaturated fatty acid content in muscle
Source: PLoS One. 2018 Nov 5;13(11):e0206615. doi: 10.1371/journal.pone.0206615 (PMC6218049; doi:10.1371/journal.pone.0206615)
Supplement: S3 Table — Log2FC is the result of transcriptome analysis, Reg is regulation, and inf is infinite. (DOCX) [file pone.0206615.s003.docx]

**S3 Table. Comparison of gene expression results between transcriptomic and qPCR data.** Log2FC is the results of transcriptome analysis, Reg is regulation, and inf is infinite.

| **Gene Name** | **log2FC** | **Reg** | **qPCR** | **Reg** |
| --- | --- | --- | --- | --- |
| *phyh* | 2.31 | up | 2.5 | up |
| *H3.3* | inf | up | 0.77 | up |
| *itsn1* | 1.63 | up | 1.94 | up |
| *beta-ketoacyl* | 11.54 | up | 710.79 | up |
| *flad1* | 5.77 | up | 11.94 | up |
| *tll2* | inf | up | 610.70 | up |
| *calm* | 7.96 | up | 5.92 | up |
| *nlrp12* | -2.01 | down | 0.73 | up |
| *setd8-a* | -5.01 | down | 2.08 | up |
| *epd-2* | -1.93 | down | 0.46 | up |
