# Supplementary material for: Comparative transcriptomics identifies genes differentially expressed in the intestine of a new fast-growing strain of common carp with higher unsaturated fatty acid content in muscle
Source: PLoS One. 2018 Nov 5;13(11):e0206615. doi: 10.1371/journal.pone.0206615 (PMC6218049; doi:10.1371/journal.pone.0206615)
Supplement: S4 Table — (DOCX) [file pone.0206615.s004.docx]

**S4 Table. Diet composition.**

| **Ingredient** | **%** |
| --- | --- |
| Fishmeal | 15 |
| Soybean meal | 17 |
| Gluten | 5 |
| Cottonseed meal | 14 |
| Multivitamin/mineral supplement | 2 |
| Wheat starch | 35 |
| Soybean oil | 5 |
| Monocalcium phosphate | 2 |
| Bentonite | 2 |
| Cellulose | 3 |
| Total | 100 |
