# Supplementary material for: Comparative transcriptomics identifies genes differentially expressed in the intestine of a new fast-growing strain of common carp with higher unsaturated fatty acid content in muscle
Source: PLoS One. 2018 Nov 5;13(11):e0206615. doi: 10.1371/journal.pone.0206615 (PMC6218049; doi:10.1371/journal.pone.0206615)
Supplement: S5 Table — BWe = body weight (g), TL = total length (mm), CF = condition factor, BWi = body width (mm), BH = body height (mm). The last row in each group contains the average values of morphometric characteristics. (DOCX) [file pone.0206615.s005.docx]

**S5 Table. Identification details and growth parameters for the 12 specimens selected for RNA-seq and fatty acid composition analyses.** BWe = body weight (g), TL = total length (mm), CF = condition factor, BWi = body width (mm), BH = body height (mm). The last row in each group contains the average values of morphometric characteristics.

| **Line** | **Sample** | **Cage** | **ID** | **PIT number** | **BWe** | **BWi** | **BH** | **TL** | **TL/BWe** | **CF** | **logBWe** |
| --- | --- | --- | --- | --- | --- | --- | --- | --- | --- | --- | --- |
| **Control** | T01 | 1 | 187 | 900108001800093 | 33.8 | 21 | 37 | 111 | 0.304505 | 2.471427 | 3.520461 |
|  | T02 | 1 | 195 | 900108001302615 | 35.1 | 22 | 38 | 115 | 0.305217 | 2.307882 | 3.558201 |
|  | T07 | 1 | 106 | 900108001800047 | 39.1 | 20 | 41 | 114 | 0.342982 | 2.639139 | 3.666122 |
|  | T08 | 1 | 98 | 900108001302623 | 44.3 | 21 | 40 | 119 | 0.372269 | 2.628832 | 3.790985 |
| average |  |  |  |  | 38.08±4.72 | 21.00±0.82 | 39.00±1.83 | 114.75±3.30 | 0.33±0.03 | 2.51±0.16 | 3.63±0.12 |
| **Selected** | T03 | 2 | 173 | 900115000072181 | 58 | 24 | 47 | 133 | 0.43609 | 2.465319 | 4.060443 |
|  | T04 | 3 | 131 | 900115000072169 | 85 | 78 | 48 | 146 | 0.582192 | 2.731243 | 4.442651 |
|  | T05 | 4 | 123 | 900115000072188 | 52.3 | 22 | 43 | 123 | 0.425203 | 2.810518 | 3.956996 |
|  | T06 | 1 | 81 | 900115000072173 | 49.3 | 22 | 42 | 127 | 0.388189 | 2.406776 | 3.897924 |
|  | T09 | 2 | 77 | 900115000072190 | 86.8 | 27 | 52 | 153 | 0.56732 | 2.423513 | 4.463607 |
|  | T10 | 2 | 66 | 900115000072155 | 38.9 | 22 | 37 | 114 | 0.341228 | 2.625639 | 3.660994 |
|  | T11 | 3 | 44 | 900115000072176 | 77 | 26 | 46 | 147 | 0.52381 | 2.424034 | 4.343805 |
|  | T12 | 4 | 1 | 900115000072153 | 64.7 | 24 | 44 | 138 | 0.468841 | 2.461881 | 4.169761 |
| average |  |  |  |  | 64±17.52 | 30.63±19.23 | 44.88±4.49 | 135.13±13.37 | 0.47±0.09 | 2.54±0.16 | 4.12±0.28 |
