# Supplementary material for: Comparative transcriptomics identifies genes differentially expressed in the intestine of a new fast-growing strain of common carp with higher unsaturated fatty acid content in muscle
Source: PLoS One. 2018 Nov 5;13(11):e0206615. doi: 10.1371/journal.pone.0206615 (PMC6218049; doi:10.1371/journal.pone.0206615)
Supplement: S6 Table — (DOCX) [file pone.0206615.s006.docx]

**S6 Table. Ct values of three tested reference genes in all samples.**

| **Reference gene** | **Selection** | **Control** |
| --- | --- | --- |
| *18s* | 17.34±0.63 | 17.27±0.18 |
| *beta actin* | 15.69±0.25 | 15.01±0.17 |
| *gapdh* | 17.94±0.38 | 18.15±0.24 |
