# Supplementary figures and images for: Comparative transcriptomics identifies genes differentially expressed in the intestine of a new fast-growing strain of common carp with higher unsaturated fatty acid content in muscle
Source: PLoS One. 2018 Nov 5;13(11):e0206615. doi: 10.1371/journal.pone.0206615 (PMC6218049; doi:10.1371/journal.pone.0206615)

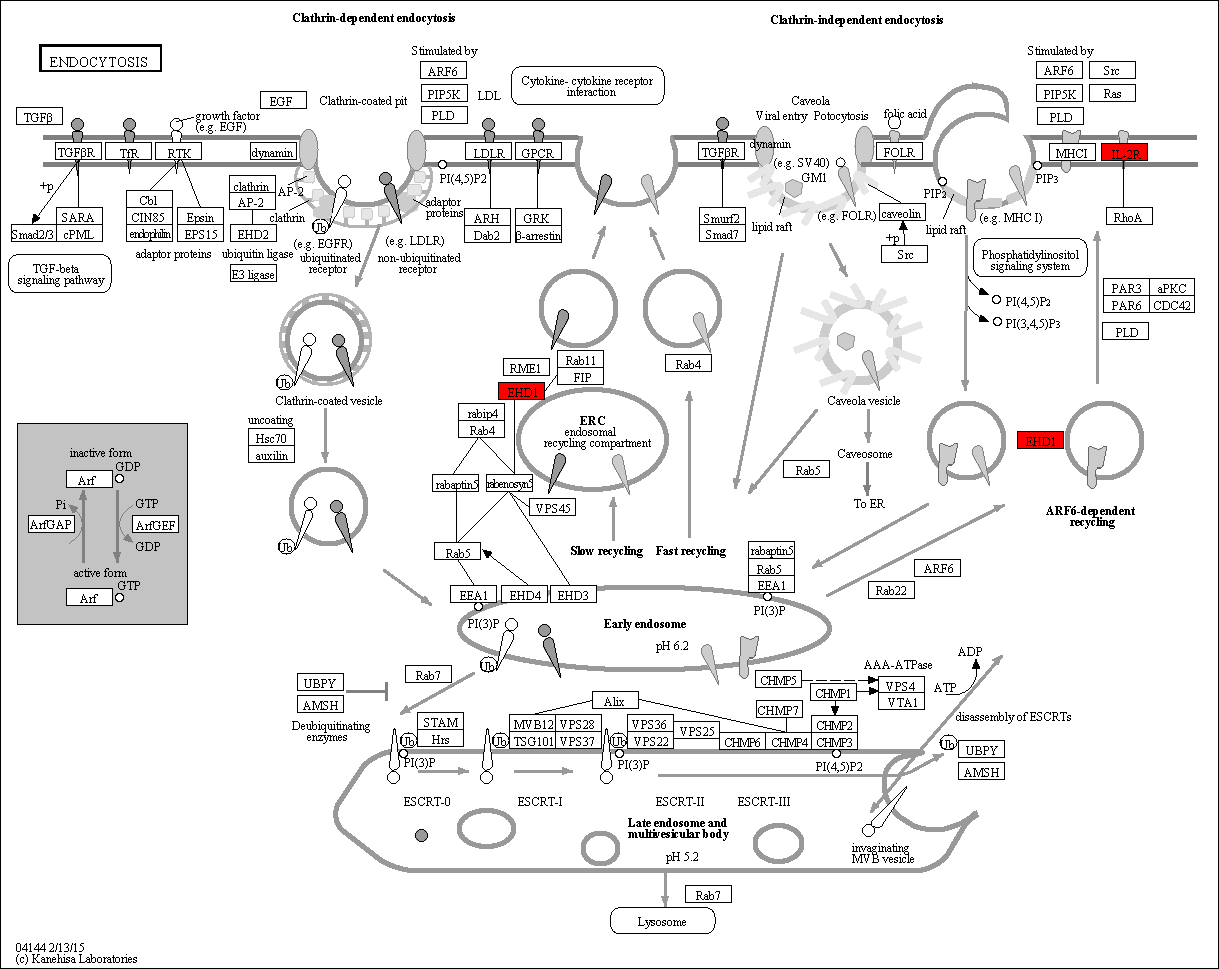

Supplement: S1 Fig — Significantly enriched genes are highlighted in red. (PNG) [file pone.0206615.s007.png]

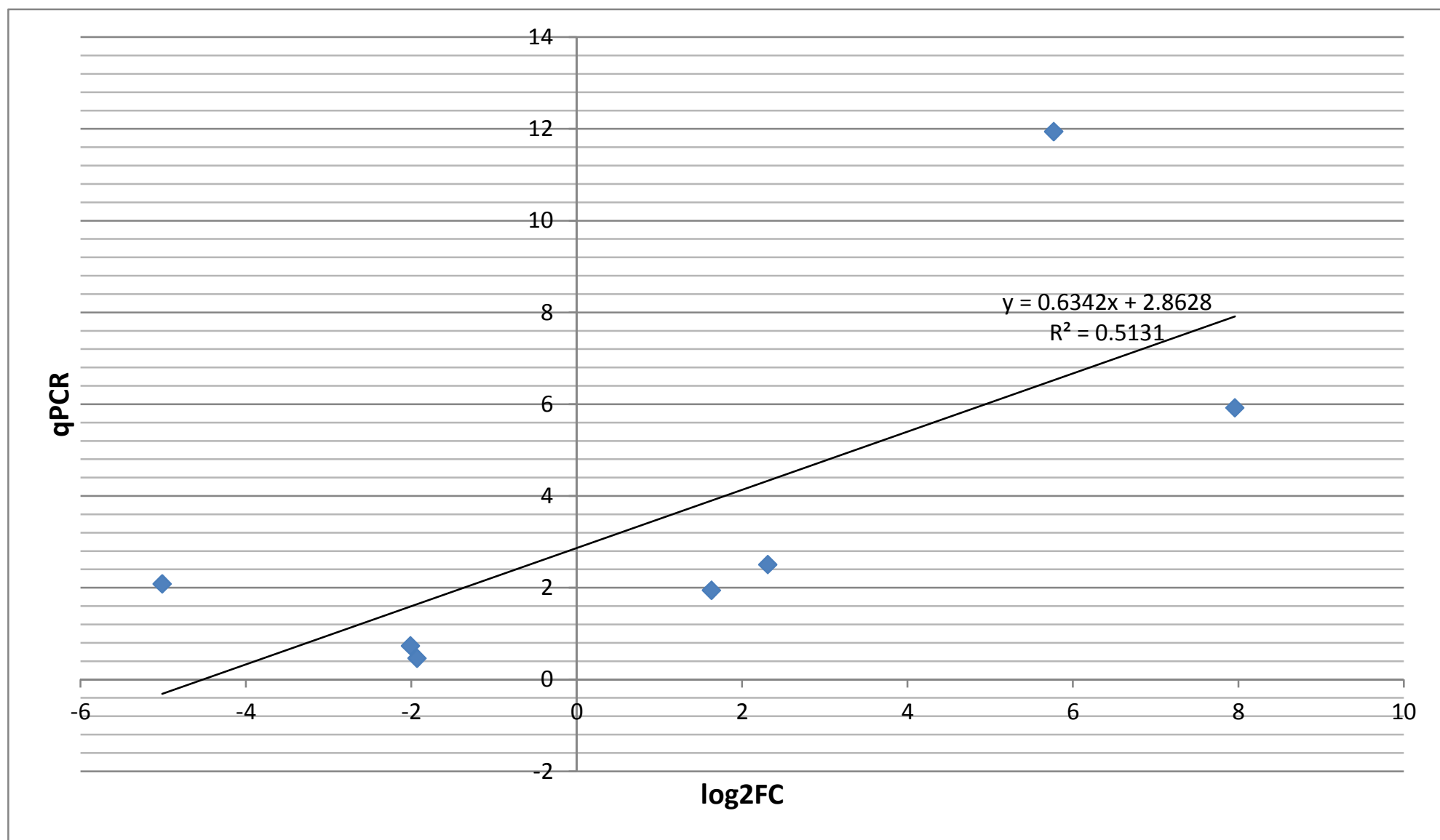

Supplement: S2 Fig — Transcriptome results are presented on the x-axis (log2FC) and qPCR on the y-axis. The following outliers were removed from the analysis: h3.3 and tll2 (inf) and novel β-ketoacyl (>700) (see S3 Table). (PDF) [file pone.0206615.s008.pdf]

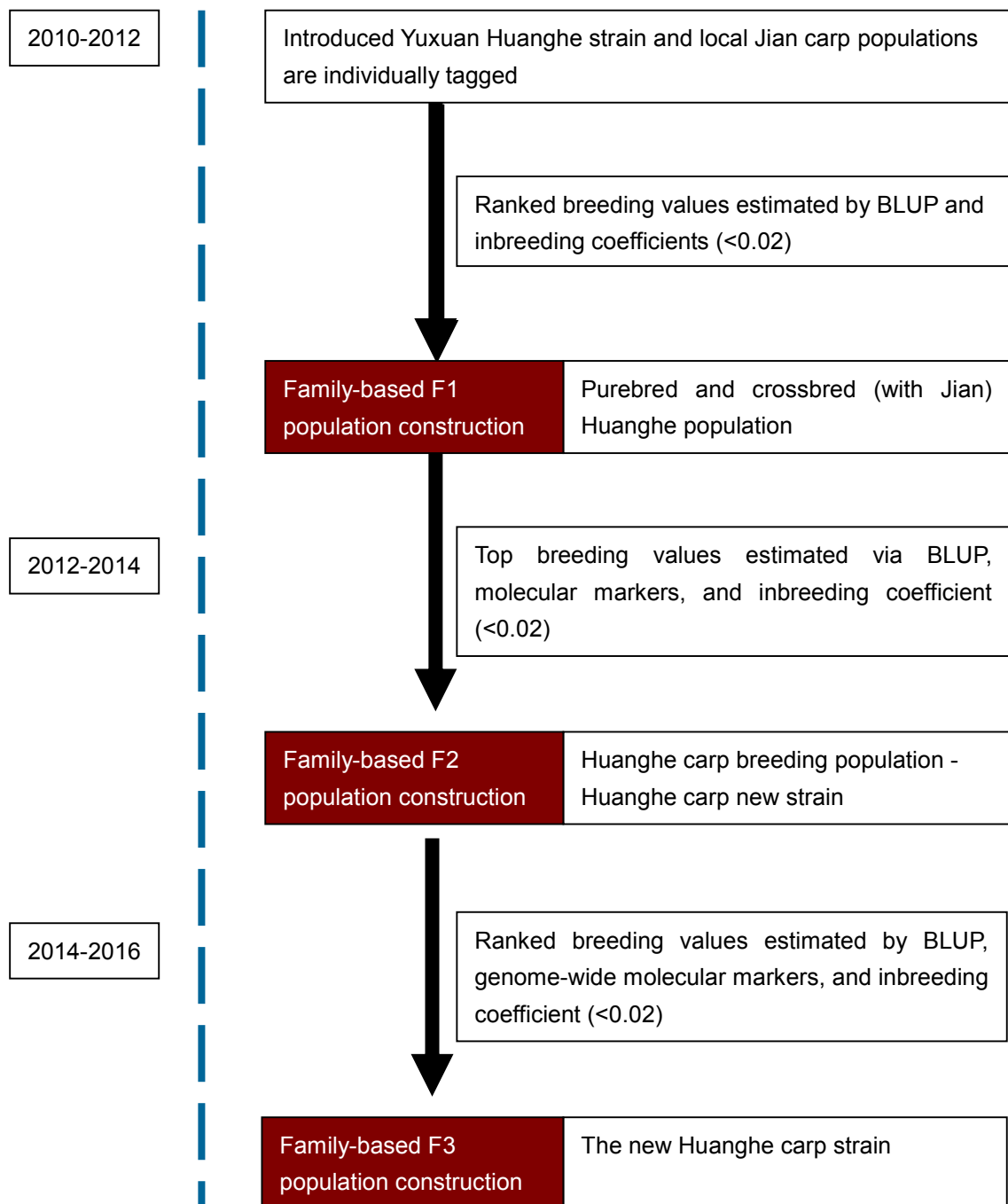

Supplement: S3 Fig — (PDF) [file pone.0206615.s009.pdf]
